# Effect of telecare on use of health and social care services: findings from the Whole Systems Demonstrator cluster randomised trial

**DOI:** 10.1093/ageing/aft008

**Published:** 2013-02-25

**Authors:** Adam Steventon, Martin Bardsley, John Billings, Jennifer Dixon, Helen Doll, Michelle Beynon, Shashi Hirani, Martin Cartwright, Lorna Rixon, Martin Knapp, Catherine Henderson, Anne Rogers, Jane Hendy, Ray Fitzpatrick, Stanton Newman

**Affiliations:** 1 The Nuffield Trust, London, UK; 2 Robert F. Wagner Graduate School of Public Service, New York University, New York, NY, USA; 3 University of East Anglia, Norwich, UK; 4 School of Health Sciences, City University, London, UK; 5 London School of Economics and Political Science, London, UK; 6 King's College London, London, UK; 7 Faculty of Health Sciences, University of Southampton; 8 Health Care Management and Policy Department, University of Surrey, Guildford, UK; 9 University of Oxford, Oxford, UK

**Keywords:** telecare, assistive technology, randomised controlled trial, administrative data, older people

## Abstract

**Objective:** to assess the impact of telecare on the use of social and health care. Part of the evaluation of the Whole Systems Demonstrator trial.

**Participants and setting:** a total of 2,600 people with social care needs were recruited from 217 general practices in three areas in England.

**Design:** a cluster randomised trial comparing telecare with usual care, general practice being the unit of randomisation. Participants were followed up for 12 months and analyses were conducted as intention-to-treat.

**Data sources:** trial data were linked at the person level to administrative data sets on care funded at least in part by local authorities or the National Health Service.

**Main outcome measures:** the proportion of people admitted to hospital within 12 months. Secondary endpoints included mortality, rates of secondary care use (seven different metrics), contacts with general practitioners and practice nurses, proportion of people admitted to permanent residential or nursing care, weeks in domiciliary social care and notional costs.

**Results:** 46.8% of intervention participants were admitted to hospital, compared with 49.2% of controls. Unadjusted differences were not statistically significant (odds ratio: 0.90, 95% CI: 0.75–1.07, *P* = 0.211). They reached statistical significance after adjusting for baseline covariates, but this was not replicated when adjusting for the predictive risk score. Secondary metrics including impacts on social care use were not statistically significant.

**Conclusions:** telecare as implemented in the Whole Systems Demonstrator trial did not lead to significant reductions in service use, at least in terms of results assessed over 12 months.

International Standard Randomised Controlled Trial Number Register ISRCTN43002091.

## Introduction

Telecare has been used for some time to support independent living for frail people. Telecare covers a range of technology, the most basic being a pendant alarm, already used by 1.5 million people in the UK. Newer forms enable ‘remote monitoring of condition or lifestyle’ [1], such as detectors for falls and bed occupancy. Unlike the pendant alarm, these newer forms of telecare gather and transfer information automatically to monitoring centres. Attention from carers is then prompted in the event of behaviour that differs from routine patterns.

The investment made so far into telecare has happened without robust evidence about its effects on use of services and associated costs [2, 3], perhaps because the focus has been on individual outcomes such as quality of life. Yet many claims have been made about the potential impact of telecare on service use [4], and if true these would have significant implications for service planning and the funding of care. In recognition of the need for evidence, in 2006 the Department of Health established the Whole Systems Demonstrator pilots of integrated care supported by technologies such as telecare [5]. There were three pilots, in Cornwall, Kent and Newham in England. The evaluation included a large randomised controlled trial of telecare [1].

The services affected could span both social and health-care sectors [6]. For example, telecare may reduce admissions to permanent residential and nursing homes, through supporting independence or by easing the burden of care for carers [7]. It might also replace face-to-face contact in domiciliary care, which would be of concern if users are socially isolated. Furthermore, telecare might enable faster response to falls, thus reducing hospital admissions [8] or facilitate faster discharge from hospital, thereby reducing the length of stay. Alternatively, telecare might uncover unmet need, increasing service use.

## Methods

### Interventions

The trial protocol has been published separately, so is only summarised here [1]. The three sites were left to design and procure their own telecare systems but all intervention participants were given a *Tunstall Lifeline Connect* or *Connect+* base unit together with a pendant alarm and up to 27 peripheral devices, assigned by local teams. These covered [9]:

- Functional monitoring, including the ‘Lifeline’ base units and pendants, bed and chair occupancy sensors, enuresis sensors, epilepsy sensors, fall detectors and medication dispensers.
- Security monitoring, including bogus caller buttons, infrared movement sensors and property exit sensors.
- Environmental monitoring, including gas, monoxide and smoke detectors, heat sensors, temperature extremes sensors and flood detectors.
- Standalone devices not linked to a monitoring centre, such as big button phones, key safes for carers and memo minders.

Data from the peripheral devices were sent to a monitoring centre via a telephone line and alerts were monitored continuously.

Telecare was compared with ‘usual care’. This reflected the existing range of health and social care services available in the areas, which might include more basic forms of telecare such as pendant alarms and smoke detectors. At the end of the 12 months of the trial, control individuals were offered telecare if they were still eligible.

### Study recruitment

All general practices in Cornwall, Kent and Newham were eligible to participate in the trial. Those that accepted the invitation to participate were randomly allocated to intervention or control groups based on a centrally administered minimisation algorithm (described in detail elsewhere [1]). Inclusion criteria for individuals required age over 18 and one or more of the following [1]:

- a minimum level of social care service (or being considered to need it);
- mobility difficulties;
- a history of falls or high risk of falling;
- cognitive impairment or confusion with a live-in or nearby carer or
- a carer facing difficulties.

People were excluded if already in receipt of telecare, unless it was only a pendant alarm or smoke detector.

Potentially eligible individuals who responded to initial contact received a ‘light-touch visit’ from project staff, where consent was taken to participate in the trial. Although individuals were not informed of their intervention or control status until after the point of consent, the extended period of recruitment meant it was not always possible to blind recruiters to practice allocations.

### Endpoints and sample size

Our primary endpoint was the proportion of people experiencing an inpatient hospital admission within 12 months (the ‘admission proportion’). The primary hypothesis was that telecare could alter the admission proportion in either direction, and the study was powered to detect a relative change of 17.5% from an assumed usual care level of 25% (based on *a priori* site estimates), at power 80% and a *P*-value of <0.05. Sample size calculations allowed for an intra-cluster correlation coefficient of 0.001, based on previous studies [10]. A total of 3,000 participants were required (25 participants from each of an assumed 120 general practices).

Secondary endpoints calculated over 12 months were

- mortality;
- proportion of people admitted to permanent residential or nursing care that was paid for at least in part by the local authority;
- number of weeks receiving domiciliary social care paid for at least in part by the local authority;
- number of inpatient hospital bed days, emergency admissions, elective admissions, admissions for falls, outpatient attendances and accident and emergency visits;
- length of inpatient hospital stays (i.e. a measure of how quickly people were discharged after being admitted to hospital);
- number of contacts with general practitioners and practice nurses and
- associated notional costs of hospital care, social care and general practice care.

Data concerning the use of these services and mortality were extracted from operational systems, linked at the person level and classified. For more information about the data sources, data linkage, variable definitions and unit costs, please see Supplementary data in *Age and Ageing* online, Appendix S1.

### Statistical approach

Individual-level data were analysed according to general practice-randomised allocations assuming a 12-month follow-up period for all participants. Baseline characteristics of intervention and control participants were compared using the ‘standardised difference’ [11], with a threshold of 10% adopted to describe meaningful differences [12].

Study endpoints were compared using three models. The first had no adjustment for baseline characteristics, whereas the second adjusted for age band, sex, ethnicity, site (Cornwall, Kent or Newham), number of chronic health conditions, an area-based deprivation score and prior service use. The third used the Combined Predictive Model score [13], which estimates the probability of an emergency hospital admission in the following 12 months based on general practice and hospital data. Where general practices did not give consent to provide data, scores were imputed based on the available hospital data using single linear imputation on the logit scale.

Practice-level clustering was accounted for using multilevel models with random effects. Logistic regression was used to estimate binary endpoints and Poisson for count endpoints. Notional costs and bed days were incremented and log transformed so that the assumptions required for subsequent ordinary least squares modelling were met.

Differences in the length of inpatient hospital stays was assessed using Cox regression to test for differences in the daily rate of discharge after admission to hospital [14]. This used random effects (frailties) to take account of clustering [15] and adjusted for the covariates in the models described above and admission method (emergency/elective).

## Results

**Figure 1.**
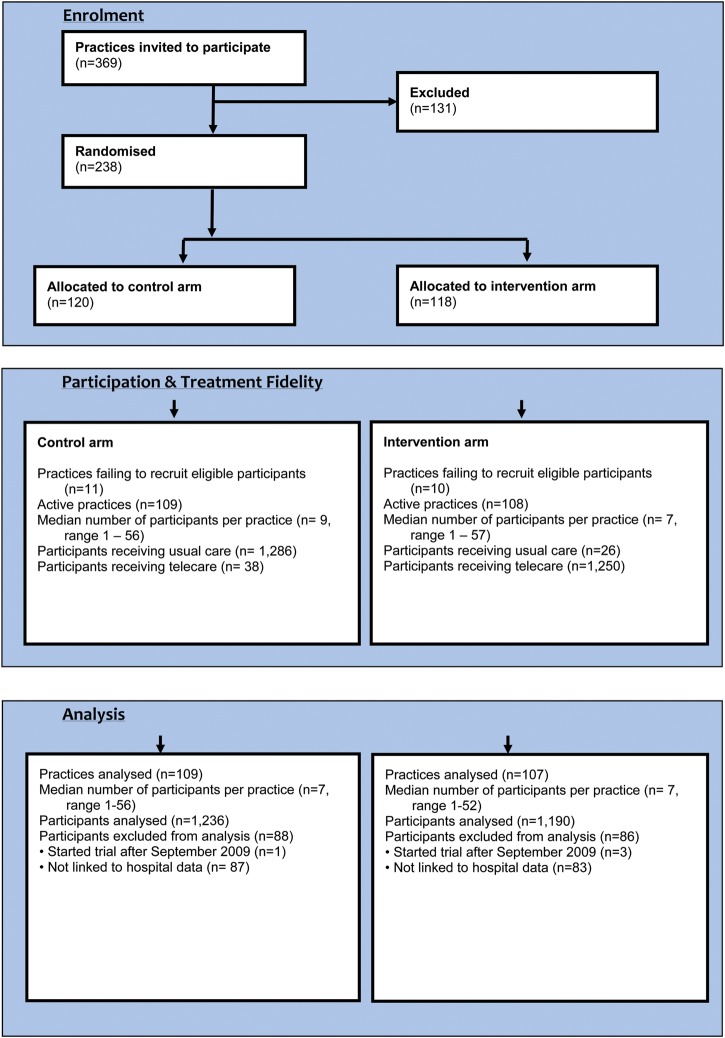
CONSORT diagram.

Figure 1 shows the flow of practice and individual recruitment and analysis. 238 practices were allocated to control or intervention groups, of which 217 ultimately supplied participants for the trial. Sites recruited 1,324 control participants and 1,276 intervention participants, with each practice recruiting an average of 12 participants. Recruitment started in July 2008 and was planned to finish in September 2009; four people who were recruited after this finish date were excluded from the current analyses. In addition, 170 participants could not be linked to administrative data on secondary care use. Overall, 1,236 control participants and 1,190 intervention participants were included in the analyses (93.3% of those recruited).

Across both groups, 64 of the participants included in the analyses did not receive their randomised allocations. A further 117 participants allocated to the intervention group received only a basic package (base unit with pendant alarm and/or smoke detector). Those allocated to the intervention group received 4.2 peripheral devices on average. Almost all (97.9%) had some form of functional monitoring, with 22.8% having falls detectors. Environmental (89.2%), standalone (46.4%) and security monitoring (10.3%) devices were received more rarely.

Intervention and control groups appeared similar at baseline, with four standardised differences >10% (Table 1). The intervention group contained more people from Cornwall, whereas the control group had more people in the least deprived areas. Further, the intervention group visited general practitioners more often than controls during the 3 months before the trial (2.30 per head compared with 2.04), with higher associated costs.

**Table 1.** Baseline characteristics of participants (data are % of people unless otherwise stated)

	Control (*n* = 1,236)	Intervention (*n* = 1,190)	Standardised difference (%)
No. of practices	109	107	
No. of participants per practice [median (range)]	7 (1–56)	7 (1–52)	
No. of long-term health conditions per person^a^ [mean (SD)]	1.1 (1.4)	1.2 (1.5)	1.2
Site			
Cornwall	37.9	43.0	10.4
Kent	33.2	32.1	−2.3
Newham	28.9	24.9	−9.1
Age [mean (SD) age in years]	75.4 (14.2)	75.4 (14.5)	0.0
<65 years	19.9	20.7	1.9
65–74 years	20.1	18.7	−3.4
75–84 years	32.4	32.2	−0.4
≥85	27.7	28.4	1.6
Female	67.5	67.6	0.2
Ethnicity			
White	75.0	77.3	5.4
Non-white	9.8	10.3	1.5
Unknown	15.2	12.4	−8.0
Area-level deprivation^b^ (mean (SD))	29.0 (14.5)	28.5 (13.9)	−3.6
First quartile	8.2	5.3	−11.7
Second quartile	14.9	18.5	9.7
Third quartile	34.5	34.9	1.0
Fourth quartile	42.4	41.2	−2.3
Service use (3 months prior to start date)			
Hospital admission	17.2	18.6	3.7
Surgery visit^c^	77.8	76.4	−3.4
Local authority social care	33.3	33.5	0.4
Emergency hospital admissions per head [mean (SD)]	0.14 (0.46)	0.18 (0.53)	7.3
Hospital bed days per head [mean (SD)]	1.86 (7.83)	2.09 (8.51)	2.7
Elective hospital admissions per head [mean (SD)]	0.09 (0.34)	0.10 (0.49)	0.9
Outpatient attendances per head [mean (SD)]	1.04 (2.38)	1.08 (2.08)	1.9
A&E visits per head [mean (SD)]	0.15 (0.48)	0.19 (0.53)	7.2
Falls admissions per head [mean (SD)]	0.02 (0.17)	0.04 (0.21)	7.4
General practitioner contacts per head^c^ [mean (SD)]	2.04 (2.34)	2.30 (2.72)	10.2
Practice nurse contacts per head^c^ [mean (SD)]	0.81 (2.03)	0.96 (2.49)	6.8
Domiciliary care weeks per head [mean (SD)]	3.82 (5.76)	3.79 (5.71)	−0.6
Care home weeks per head [mean (SD)]	0.07 (0.90)	0.02 (0.43)	−6.9
Cost over 3 months prior to trial			
Hospital tariff cost per head [£ mean (SD)]	601 (1719)	628 (1789)	1.6
GP surgery cost per head^c^ [£ mean (SD)]	91 (110)	104 (124)	11.3
Social care cost per head [£ mean (SD)]	997 (1512)	963 (1455)	−2.3
Combined predictive model score^d^	0.24 (0.19)	0.24 (0.18)	0.2
Low risk	18.8	17.1	−4.3
Moderate risk	34.0	34.7	1.3
High risk	39.0	40.5	3.1
Very high risk	8.2	7.7	−1.9

SD, standard deviation.

^a^Count of long-term health conditions is based on inpatient hospital data.

^b^*n* = 1,227 for the control group, *n* = 1,188 for the intervention group. First quartile is least deprived, fourth quartile is most deprived. Deprivation scores are based on Index of Multiple Deprivation 2007.

^c^*n* = 1,032 for the control group, *n* = 889 for the intervention group

^d^*n* = 1,090 for the control group, *n* = 1,044 for the intervention group. Risk categories denote top proportions of site population: very high risk (0.5%), high risk (0.5–5%), moderate risk (5–20%) and low risk (20–100%).

Of intervention participants, 46.8% were admitted to hospital within the 12 months of the trial, compared with 49.2% of controls. This corresponded to an absolute difference of −2.4% or a relative difference of −4.8% (95% CI: −12.9 to 3.2%) (Table 2). This difference was not statistically significant in the unadjusted analysis (odds ratio: 0.90, 95% CI: 0.75–1.07, *P* = 0.211). It reached significance when adjusting for baseline characteristics (*P* = 0.042) but not when adjusting for the combined predictive model score (*P* = 0.202). More information is available in the Supplementary data online, Appendix S2.

**Table 2.** Service use and mortality during trial (unadjusted for clustering and covariates)

	Control (*n* = 1,236)	Intervention (*n* = 1,190)	Absolute difference (95% CI)	Percentage (relative) difference (95% CI)
Admission proportion (%)	49.2	46.8	−2.4 (−6.36 to 1.59)	−4.8 (−12.9 to 3.2)
Mortality (%)	8.9	8.7	−0.24 (−2.50 to 2.01)	−2.7 (−28.1 to 22.6)
Emergency hospital admissions per head	0.57 (1.02)	0.65 (1.36)	0.08 (−0.01 to 0.18)	14.7 (−2.1 to 31.4)
Elective hospital admissions per head	0.41 (1.02)	0.38 (1.10)	−0.04 (−0.12 to 0.05)	−8.6 (−29.0 to 12.1)
Outpatient attendances per head	3.80 (7.19)	3.92 (7.12)	0.12 (−0.45 to 0.69)	3.2 (−11.8 to 18.2)
A&E visits per head	0.70 (1.18)	0.72 (1.60)	0.02 (−0.09 to 0.14)	3.4 (−12.6 to 19.4)
Falls admissions per head	0.11 (0.41)	0.14 (0.53)	0.02 (−0.01 to 0.06)	21.9 (−12.0 to 55.9)
Hospital bed days per head	8.48 (20.65)	8.65 (21.42)	0.17 (−1.50 to 1.85)	2.0 (−17.7 to 21.8)
General practitioner contacts per head^a^	6.63 (8.00)	6.72 (8.10)	0.09 (−0.55 to 0.73)	1.4 (−8.3 to 11.1)
Practice nurse contacts per head^a^	3.21 (7.80)	2.80 (5.90)	−0.41 (−0.96 to 0.15)	−12.7 (−29.9 to 4.6)
Proportion admitted to permanent residential or nursing care (%)	3.2	3.1	0.00 (−0.01 to 0.01)	−1.5 (−45.4 to 42.5)
Domiciliary care weeks per head	15.36 (22.44)	15.41 (22.59)	0.05 (−1.74 to 1.85)	0.3 (−11.3 to 12.0)
Hospital tariff cost per head (£)	2,604 (4,707)	2,846 (5,427)	242 (−162 to 647)	9.3 (−6.2 to 24.8)
GP surgery cost per head (£)^a^	315 (400)	305 (363)	−10 (−40 to 20)	−3.2 (−12.9 to 6.5)
Social care cost per head (£)	4,287 (6,184)	4,210 (6,070)	−77 (−565 to 411)	−1.8 (−13.2 to 9.6)

Data are mean (SD) unless stated otherwise.

^a^*n* = 1,032 for the control group, *n* = 889 for the intervention group.

A similar proportion of intervention and control participants were admitted to permanent residential and nursing care during the 12 months (3.1 and 3.2%, respectively). The odds ratio for admission had a very wide confidence interval (unadjusted odds ratio: 0.95, 95% CI: 0.57–1.59, *P* = 0.860). There were also no significant differences in the number of weeks receiving domiciliary social care between groups (unadjusted incidence rate ratio: 1.03, 95% CI: 0.73–1.44, *P* = 0.862). General practitioner contacts were significantly higher among intervention than controls in the unadjusted analysis (incidence rate ratio: 1.18, 95% CI: 1.01–1.38, *P* = 0.033), though this did not persist after adjusting for the prior differences in use (*P* = 0.064). There were no significant differences between groups in the cost associated with hospital care and social care; it was not possible to adjust general practice costs for prior differences in general practice use. Mortality rates were not significantly different between groups.

There were no significant differences in lengths of hospital stays (hazard ratio from Cox regression, 1.005 when adjusting for the combined model score and admission method, 95% CI: 0.922–1.095, *P* = 0.91, based on the 2,436 admissions that occurred). Although log–log survival curves indicated that the proportional hazards assumption was not met, significant differences were not found when using time-dependent covariates.

## Discussion

No randomised studies of telecare exist on a comparable scale. No convincing evidence of effect on hospital admissions was found of the magnitude that was judged relevant at the outset of the study. Differences in the proportion of individuals admitted to hospital were detected with one form of case-mix adjustment, but these were not consistent across different forms of adjustment.

No impacts were indicated in rates of hospital use, length of inpatient hospital stay or admissions to residential or nursing care. Higher levels of general practitioner contacts were detected among intervention than control participants, but differences appeared to exist before the trial and adjusting for prior use removed the significance of results.

This study forms one part of the Whole Systems Demonstrator evaluation and focuses on system outcomes. Other evaluation themes will assess effects on quality of life for a subsample of participants and address the views of service users and carers. A cost-effectiveness study will estimate the cost of the telecare intervention (not included in the current analysis) and capture services such as community nursing, social work and paramedics. It will also reflect the number of hours of domiciliary care received, rather than the period over which domiciliary care was received, as in the current study. The Whole Systems Demonstrator project also examined a system of remote patient monitoring (‘telehealth’) for a separate population in the same sites who had long-term health conditions. This has reported separately [16].

In this study, telecare consisted of devices aimed at remote, automatic and passive monitoring, and it was compared with usual care that may include more basic telecare such as pendant alarms. Telecare should be considered as just one element within the system in which it is used. All participants (including controls) could have benefited from the wider service redesign associated with the trial [1]. Therefore the study assessed the added value of telecare over and above the effects of this wider service redesign. Although the multisite nature of the trial adds to generalisability, telecare might have different effects in other settings or when implemented differently.

A very low proportion of controls (3.2%) had been admitted to permanent residential and nursing care by 12 months. Thus it appears that the either the intervention was not applied to a population at high risk of admission to a care home, or that high-risk individuals may have been considered unsuitable for enrolment; it may be that any benefits on care home admissions only materialise over longer time periods or in specific subgroups of users.

Administrative data were available for almost all participants (93.3%); however, the sites could not provide consistent information for us to test for differences between groups in social care needs. Despite this, administrative data on social care use provide new opportunities for research [17, 18].

The trial was designed to minimise bias [19] but the complexity of the trial meant it could not be fully blinded, as recruiters knew practice allocations in some cases. We were reassured by the similarity of cluster sizes between intervention and control groups, though there were differences in prior general practice use that were taken into account using case-mix adjustment. The ‘intention to treat’ approach to analysis preserved randomisation, though may have produced conservative estimates as a small number of control participants received telecare and vice versa.

The target number of participants for this study was 3,000, but data for only 2,426 people were available. Thus, despite the large numbers of individuals recruited, this study was potentially underpowered to detect the relative difference in the hospital admission proportion which was thought to be relevant at the outset of the study (17.5%). However, as the −4.8% difference observed was notably smaller than that considered meaningful and had a 95% confidence interval of −12.9 to 3.2%, even the largest likely true effect of the intervention (−12.9%) does not reach the 17.5% level. Since a larger study would, all other factors being equal, simply have a narrower confidence interval around the study estimates, the non-significant result does not reflect the fact that the study was likely to have been underpowered. We note, however, that we could not rule out a reduction as large as 12.9%.

We conclude that telecare did not significantly alter rates of health or social care service use or mortality among a population with social care needs over 12 months. This is the first large randomised study to test for these impacts and the findings will have implications for resource use and planning. Decision-making should take account of forthcoming results in relation to the quality of life, carer outcomes and experience.

**Key points**

- We conducted a large randomised controlled trial of telecare, with 2,600 participants from three areas of England.
- In this trial, telecare did not significantly alter rates of health or social care service use or mortality over 12 months.
- Decisions should also reflect findings from other strands of this evaluation, such as those relating to quality of life.

## Supplementary data

Supplementary data mentioned in the text is available to subscribers in *Age and Ageing* online.

## Authors' contributions

A.S. led the collection of administrative datasets, analysis and manuscript drafting. M.B., J.D. and J.B. took part in the original design of this element of the work and contributed to the analysis. Additionally, H.D., S.H., M.K., A.R., R.F., J.H. and S.N. were involved in the development of the study protocol for the overall project. S.N. was principal investigator of the Whole Systems Demonstrator trial and H.D. was statistical adviser and guarantor of the statistical robustness of the overall project. M.B., M.C., S.H. and L.R. co-ordinated the daily implementation of the trial protocol and maintained trial participants' data. C.H. provided input to the analysis. All authors reviewed the manuscript. The Whole Systems Demonstrator Evaluation Team contributed to periodic discussions of the data collected for this study during team meetings, and commented on interim documents produced during the study.

## Conflicts of interest

All authors declare support from the Department of Health and the University College London Hospitals and University College London; several authors have undertaken evaluative work funded by government or public agencies, but these have not created competing interests; no other relationships or activities that could appear to have influenced the submitted work.

## Ethical approval

The study was approved by Liverpool research ethics committee (reference 08/H1005/4).

## Funding

The study was funded by the Department of Health in England. The Department of Health reviewed the protocol for the study and provided project manager support for the implementation of telecare. The University College London Hospitals and University College London were the Whole Systems Demonstrator study sponsors. Their role as sponsors was to ensure that the study was conducted in accordance with the Research Governance Framework for Health and Social Care (2nd edition, April 2005) and to confirm that arrangements were in place for the initiation, management, monitoring and financing of the trial.

## Supplementary Material

Supplementary Data
