# Supplementary material for: Effect of telecare on use of health and social care services: findings from the Whole Systems Demonstrator cluster randomised trial
Source: Age Ageing. 2013 Feb 25;42(4):501–8. doi: 10.1093/ageing/aft008 (PMC3684109; doi:10.1093/ageing/aft008)
Supplement: Supplementary Data [file supp_42_4_501__index.html]

Effect of telecare on use of health and social care services: findings from the Whole Systems Demonstrator cluster randomised trial — Supplementary Data 

# Effect of telecare on use of health and social care services: findings from the Whole Systems Demonstrator cluster randomised trial

## Supplementary Data

Supplementary Data

**Files in this Data Supplement:**

- Supplementary Data - Doc file
