# Supplementary material for: Effect of telecare on use of health and social care services: findings from the Whole Systems Demonstrator cluster randomised trial
Source: Age Ageing. 2013 Feb 25;42(4):501–8. doi: 10.1093/ageing/aft008 (PMC3684109; doi:10.1093/ageing/aft008)
Supplement: Supplementary Data [file supp_aft008_aft008supp.doc]

Supplementary Data

**Appendix 1: data extraction, linkage and classification**

***Data sources and linkage approach***

Participants were linked at the person level to data for inpatient and outpatient secondary activity sourced from Hospital Episode Statistics, a national data warehouse for England. Participants were linked by the NHS Information Centre for health and social care, a trusted third party that was the only organisation to have access to both patient identifiers and data for secondary care activity. A linked mortality file provided data for all deaths occurring in and out of hospital. In addition, participants were linked to local commissioning datasets on visits to accident and emergency departments. These data were “pseudonymised” before being transferred to the research team, so that patient identifiable fields were removed and a unique patient identifier (the “NHS number”) encrypted. The encrypted NHS number was then used to link activity to trial participants.

All general practices participating in the trial were asked to share person-level data for the whole of the adult practice population, relating to registration and encounter dates, diagnoses, test results and prescriptions. Where possible, data were extracted remotely and out-of-hours. Alternatively, staff in the four Primary Care Trusts visited the practices with MiQuest queries. In either case, data were pseudonymised and linked using the encrypted NHS number.

Social care data related to “service elements” (items of service received for defined periods of time) funded at least in part by local authorities. As unique patient identifiers do not exist across social care, alternative identifiers were attributed to social care data based on date of birth, initials and gender. In two of the sites, linkage of social care data was validated by a hand-search by staff in the local authorities.

***Variable definitions***

Staff in the local authorities helped us identify service elements related to domiciliary care, direct payments and permanent residential and nursing care. Inpatient activity was limited to ordinary admissions and excluded regular ward attendances, maternity events and transfers. Admissions were classified as emergency or elective based on the recorded method of admission. Bed days included stays following both emergency and elective activity; same day admissions and discharges assigned a length of 1 bed day. Falls-related admissions were identified by an ICD-10 code in the range W00-W19 in any diagnosis field. Outpatient activity was restricted to appointments that were attended. General practice contacts were divided into those with general practitioners and practice nurses and included all recorded patient encounters at all locations.

The comparisons of general practice use were limited to those patients with a record of registration at practices for which data were available. Periods of practice data were excluded if patterns of contacts were anomalous and indicated incomplete data. A continuous registration was required for the twelve months of the trial and the two previous years for an individual to be included in analyses of general practice use.

***Results of data extraction***

Primary care data were extracted for 209 general practices. Consequently, Combined Predictive Model scores were available for 1,090 control and 1,044 intervention patients (88.0% of the total included). Scores were imputed for the remainder. Analysis of GP use was restricted to 1,921 participants with a record of continuous registration (79.2%). Included participants were slightly older on average than those excluded (mean age 75.7 versus 74.2), more likely to come from the two most deprived quintiles (43.5% versus 29.9%) and more likely to live in Newham (29.2% versus 18.4%).

A total of 15,439 social care service elements were identified from the social care data sets using the constructed pseudonymous identifiers. 1,910 participants had at least one recorded service element (78.7%) and 10,436 service elements were active at some point during the trial period, of which 5,000 were for home care, direct payments or permanent residential or nursing care. In the two sites for which mappings were checked by hand (which provided a total of 2,593 service elements), 2,344 service elements were confirmed (90.4%). The manual mappings identified an additional 40 service elements not included in the main analysis.

***Unit costs***

We applied notional unit costs to weight the recorded levels of activity and produce summary metrics for the impact of telecare on service use. As unit costs were not adjusted for the regional costs of providing care, they produced a nationally-standardised activity measure which allowed a robust comparison of the overall magnitude of care received for intervention and control participants:

- Social care costs were calculated for domiciliary care and permanent residential and nursing care, before contributions made by clients. Costs for nursing care included the amount paid by the NHS.
- Hospital costs included inpatient and outpatient care. Unit costs were based on the mandatory and indicative Payments by Results (PbR) tariffs 2008/9,[1] which describe payments made by commissioners of care. Although all activity was included in the analysis of rates of hospital use, we did not cost activity not covered by the tariffs, such as mental health, critical care, cystic fibrosis, high cost drugs and outpatient physiotherapy, as well as accident and emergency visits.
- General practice costs reflected numbers of contacts with general practitioners and practice nurses.

The unit costs are described in Table A1.

**Table A**1: Assumed unit costs

| **Service group** | **Unit cost** | **Unit** | **Source** |
| --- | --- | --- | --- |
| Inpatient admission | Median £1,679, range £217-£38,299. | Admission | 2008/9 Payment by Results tariffs |
| Outpatient attendance | Median £96, range £46-£5,885 | Attendance | 2008/9 Payment by Results tariffs |
| General practitioner contact | £32 | Consultation | PSSRU 2010 unit costs for consultation with principal GP |
| Practice nurse contact | £12 | Contact | PSSRU 2010 unit costs |
| Domiciliary care | £17.10 | Hour | National average across all LA & other provision from PSSEX 2010/11 for adults and older people. Assumed average of 2.1 hours per day. |
| Residential care | £528.50 | Week | National average across all LA & other provision from PSSEX 2010/11 for older people |
| Nursing care | £645.90 | Week | National average across all LA & other provision from PSSEX 2010/11 for older people + £108.70 NHS nursing contribution cost |

**Figure A1: Unadjusted trends in use of health and social care**


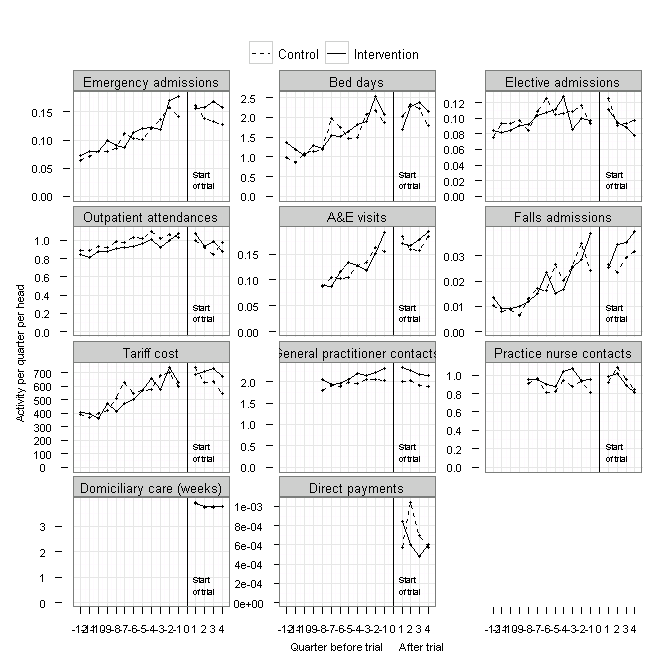


**References**

Department of Health. Payment by Results: guidance and tariff for 2008-09. 2007.

**Appendix 2**

**Table B1: Results of mixed models (including case mix adjustment)**

| **Endpoint (interpretation)** | **Model** | **Estimate (95% CI}** | **p** |
| --- | --- | --- | --- |
| Admission proportion (odds ratio) | Unadjusted | 0.90 (0.75 to 1.07} | 0.211 |
|  | Adjusted | 0.83 (0.69 to 0.99} | 0.042 |
|  | Combined Model adjusted | 0.89 (0.74 to 1.07} | 0.202 |
| Mortality (odds ratio) | Unadjusted | 0.96 (0.70 to 1.30} | 0.779 |
|  | Adjusted | 0.93 (0.69 to 1.25} | 0.624 |
|  | Combined Model adjusted | 0.96 (0.71 to 1.31} | 0.812 |
| Emergency admissions (incidence rate ratio) | Unadjusted | 1.04 (0.87 to 1.25} | 0.666 |
|  | Adjusted | 0.99 (0.86 to 1.15} | 0.922 |
|  | Combined Model adjusted | 1.05 (0.90 to 1.24} | 0.519 |
| Elective admissions (incidence rate ratio) | Unadjusted | 0.89 (0.71 to 1.13} | 0.342 |
|  | Adjusted | 0.84 (0.67 to 1.05} | 0.130 |
|  | Combined Model adjusted | 0.90 (0.71 to 1.14} | 0.369 |
| Outpatient attendances (incidence rate ratio) | Unadjusted | 1.03 (0.86 to 1.23} | 0.750 |
|  | Adjusted | 1.08 (0.91 to 1.28} | 0.375 |
|  | Combined Model adjusted | 1.03 (0.85 to 1.26} | 0.748 |
| Accident and emergency visits (incidence rate ratio) | Unadjusted | 0.99 (0.82 to 1.20} | 0.913 |
|  | Adjusted | 0.96 (0.84 to 1.10} | 0.569 |
|  | Combined Model adjusted | 1.01 (0.85 to 1.20} | 0.933 |
| Falls admissions (incidence rate ratio) * | Unadjusted | 1.07 (0.78 to 1.47} | 0.664 |
|  | Combined Model adjusted | 1.07 (0.78 to 1.47} | 0.662 |
| Bed days (difference in geometric means) | Unadjusted | -0.24 (-1.18 to 0.81} | 0.631 |
|  | Adjusted | -0.46 (-1.24 to 0.40} | 0.283 |
|  | Combined Model adjusted | -0.21 (-1.10 to 0.79} | 0.667 |
| General practitioner contacts (incidence rate ratio)** | Unadjusted | 1.18 (1.01 to 1.38} | 0.033 |
|  | Adjusted | 1.10 (0.99 to 1.21} | 0.064 |
|  | Combined Model adjusted | 1.19 (1.02 to 1.39} | 0.023 |
| Practice nurse contacts (incidence rate ratio)** | Unadjusted | 0.93 (0.69 to 1.24} | 0.612 |
|  | Adjusted | 0.95 (0.77 to 1.16} | 0.585 |
|  | Combined Model adjusted | 0.93 (0.69 to 1.25} | 0.640 |
| Proportion admitted to permanent residential or nursing care (odds ratio)* | Unadjusted | 0.95 (0.57 to 1.60} | 0.860 |
|  | Combined Model adjusted | 0.96 (0.57 to 1.59} | 0.863 |
| Domiciliary care (weeks) (incidence rate ratio) | Unadjusted | 1.03 (0.73 to 1.44} | 0.862 |
|  | Adjusted | 1.05 (0.91 to 1.21} | 0.519 |
|  | Combined Model adjusted | 1.03 (0.74 to 1.44} | 0.845 |
| Hospital tariff costs (difference in geometric means) | Unadjusted | 190 (-504 to 1113} | 0.627 |
|  | Adjusted | -150 (-701 to 561} | 0.646 |
|  | Combined Model adjusted | 175 (-506 to 1078} | 0.649 |
| GP surgery cost (difference in geometric means)*, ** | Unadjusted | 81 (12 to 165} | 0.019 |
|  | Combined Model adjusted | 69 (27 to 117} | 0.001 |
| Social care cost (difference in geometric means)* | Unadjusted | 361 (-1779 to 4328} | 0.796 |
|  | Combined Model adjusted | 355 (-1733 to 4150} | 0.793 |

* One of the models did not converge

** n=1,032 for control group, n = 889 for intervention group
